# Computed Tomography-Derived Bronchial Wall Indices in Cats with Clinical and Serological Features Compatible with Heartworm-Associated Respiratory Disease

**DOI:** 10.3390/ani16111586

**Published:** 2026-05-23

**Authors:** Sara Nieves García-Rodríguez, Jorge Isidoro Matos, J. Alberto Montoya-Alonso, Laín García-Guasch, Eva Mohr-Peraza, Elena Carretón

**Affiliations:** 1 Internal Medicine, Faculty of Veterinary Medicine, Research Institute of Biomedical and Health Sciences (IUIBS), University of Las Palmas de Gran Canaria, 35016 Las Palmas de Gran Canaria, Spain; saranieves.garcia@ulpgc.es (S.N.G.-R.); alberto.montoya@ulpgc.es (J.A.M.-A.); lain.garcia@ivcevidensia.es (L.G.-G.); eva.mohr@ulpgc.es (E.M.-P.); elena.carreton@ulpgc.es (E.C.); 2 IVC Evidensia Hospital Veterinari Molins, IVC Evidensia Hospital Veterinaria del Mar, 08005 Barcelona, Spain

**Keywords:** Heartworm-Associated Respiratory Disease, *Dirofilaria immitis*, feline heartworm disease, dirofilariosis, computed tomography, bronchial wall remodeling, airway remodeling, bronchial wall-to-pulmonary artery ratio, thoracic imaging

## Abstract

Heartworm-Associated Respiratory Disease (HARD) is an early manifestation of feline dirofilariosis caused by immature *Dirofilaria immitis* stages reaching the lungs and inducing marked inflammatory airway lesions. This study quantified computed tomography (CT)-derived bronchial wall remodeling in cats with clinical and serological features compatible with HARD using the bronchial wall-to-bronchus (BW/B) and bronchial wall-to-pulmonary artery (BW/A) ratios. Twenty-seven client-owned cats were prospectively included: 19 cats with lower-airway clinical signs and *D. immitis* antibody seropositivity, considered compatible with HARD, and 8 asymptomatic seronegative cats that underwent CT for non-cardiorespiratory clinical indications. All underwent thoracic CT under a standardized anesthetic protocol. Bronchial lumen diameter, total bronchial diameter, and pulmonary artery diameter were measured in cranial and caudal lung regions, and bronchial wall thickness was calculated to derive BW/B and BW/A. Cats compatible with HARD showed significantly increased bronchial wall thickness and higher BW/B and BW/A ratios across all evaluated lung lobes, supporting diffuse bronchial remodeling. BW/A was the only index showing a significant area-by-group interaction, suggesting a possible regional distribution pattern of bronchial wall remodeling in affected cats. Measurement repeatability and reproducibility were high overall. CT-derived bronchial wall indices, particularly BW/A, may provide an objective complementary tool for characterizing airway involvement in cats with clinical and serological profiles compatible with HARD. Because antibody seropositivity indicates exposure rather than confirmed active infection, and because of the small control group, absence of respiratory disease comparators, and lack of histopathological validation, these findings should be considered preliminary and descriptive.

## Simple Summary

Cats can develop respiratory disease after exposure to heartworm (*Dirofilaria immitis*), even in the absence of adult worms. This early condition, known as Heartworm-Associated Respiratory Disease (HARD), may cause coughing, rapid breathing, and breathing difficulty because it mainly affects the small airways and lung tissue. Diagnosing HARD is challenging, since routine heartworm tests may be negative and thoracic radiographs may show only nonspecific changes. In this study, we used computed tomography (CT) to assess bronchial wall thickening in cats with respiratory signs and heartworm antibody seropositivity, as compared with seronegative cats. We calculated two ratios relating bronchial wall thickness to the bronchial diameter (BW/B) and to the diameter of the adjacent pulmonary artery size (BW/A). Cats compatible with HARD showed thicker bronchial walls and higher values for both ratios throughout the lungs, indicating diffuse airway remodeling. The BW/A ratio was especially informative because it identified regional differences in affected cats that were not detected by BW/B. These quantitative CT measurements may provide an objective complementary approach for characterizing airway involvement in cats compatible with HARD and may provide complementary descriptive information regarding airway involvement, although their direct clinical applicability and diagnostic value remain to be established.

## 1. Introduction

Heartworm-Associated Respiratory Disease (HARD) is a clinical syndrome associated with infection of cats with immature (larval) stages of *Dirofilaria immitis* [1]. In contrast to chronic feline heartworm disease caused by adult worms, HARD results from the arrival and subsequent death of immature parasites within the pulmonary vasculature and parenchyma, eliciting a marked inflammatory reaction [1,2,3,4]. This inflammatory process can cause pulmonary parenchymal injury and typically presents as lower-airway clinical signs such as cough, tachypnea, and respiratory distress. Importantly, even in the absence of adult worms, exposure to immature *D. immitis* may induce clinically relevant vascular and bronchial changes [5,6,7].

Diagnosing HARD is challenging because adult parasites are usually absent; consequently, antigen testing and echocardiography have limited diagnostic utility [8,9]. Diagnosis therefore relies on the integration of clinical history, respiratory signs, serologic evidence of exposure (antibody positivity) and thoracic imaging findings, particularly radiographic abnormalities suggestive of pulmonary involvement [5,7,8,9,10]. However, conventional thoracic radiography may be insufficiently sensitive to detect subtle or early structural changes affecting the bronchial and vascular compartments.

Advanced imaging—especially computed tomography (CT)—is increasingly being used to evaluate feline heartworm disease [1,11]. In cats infected with adult *D. immitis*, CT studies have described characteristic findings including right-sided cardiac remodeling, enlargement and tortuosity of the pulmonary arteries, increased pulmonary arterial luminal diameter, and lesions consistent with pulmonary thromboembolism [1,6,11,12,13]. These reports underscore the value of CT for detailed assessment of cardiopulmonary structures beyond the capabilities of conventional radiography.

In addition to descriptive findings, CT-derived quantitative indices have been proposed to objectively characterize parasite-induced pulmonary remodeling. Experimental studies in cats infected with adult *D. immitis* introduced morphometric parameters such as the pulmonary artery–to-bronchial lumen (BA) ratio to detect vascular and bronchial remodeling [11]. More recent research in cats with respiratory signs and seropositivity to *D. immitis*, compatible with HARD, extended this approach by evaluating bronchial, arterial, and venous relationships across all lung lobes [14]. That study suggested that indices such as BA, bronchial-to-pulmonary vein (BV), and pulmonary vein-to-pulmonary artery (PV/PA) ratios may help characterize bronchial and vascular remodeling associated with the inflammatory response linked to exposure to *D. immitis* [14]. However, these bronchial and vascular abnormalities are not necessarily specific to HARD and may overlap with those observed in other feline airway diseases [6,7]. Therefore, the diagnostic specificity of CT-derived markers for distinguishing HARD from other causes of bronchial inflammation remains to be established.

Despite these advances, bronchial wall thickness has not been specifically evaluated by CT in any parasitic stage of feline heartworm. In particular, the bronchial wall-to-bronchial diameter (BW/B) and bronchial wall-to-pulmonary artery (BW/A) ratios have not yet been described. Because normal feline bronchial structures are small [15], direct CT measurement of bronchial wall thickness can be technically challenging and prone to variability. For this reason, indirect measurement approaches based on normalized ratios have been proposed and successfully applied in clinically asthmatic cats [16]. These indices provide an objective assessment of bronchial wall remodeling and may facilitate detection of wall alterations associated with chronic inflammatory processes, potentially including those induced by *D. immitis* infection.

Therefore, the aim of this study was to quantify bronchial wall remodeling on CT in cats with lower-airway clinical signs and *D. immitis* antibody seropositivity, a profile considered compatible with HARD, using the BW/B and BW/A ratios, and to compare these indices with those of asymptomatic seronegative cats. Importantly, antibody seropositivity was interpreted as evidence of exposure and immune response, not as definitive confirmation of active infection or of the presence of immature stages in the pulmonary vasculature at the time of CT examination. Accordingly, this study was designed to describe CT-derived bronchial wall indices in a HARD-compatible clinical context, rather than to establish CT features specific to or diagnostic for HARD.

## 2. Materials and Methods

### 2.1. Animals

A total of 27 client-owned cats underwent thoracic CT and were assigned to two groups. Group A included 19 cats with lower-airway clinical signs (cough, tachypnea, and/or respiratory distress) that were seropositive for *D. immitis* antibodies by indirect ELISA. Antibody seropositivity was interpreted as evidence of exposure to *D. immitis* and immune response, but not as definitive confirmation of active infection or of a homogeneous disease stage. Group B included 8 cats that were clinically asymptomatic for respiratory and cardiovascular disease, seronegative for *D. immitis*, and underwent CT for non–cardiorespiratory indications (e.g., trauma or neurologic disorders). These cats were therefore used as a clinically selected seronegative comparison group and should not be regarded as healthy volunteer controls.

Signalment (age, breed, sex, and body weight) and relevant medical history were recorded. Inclusion criteria were consistent with those previously described [14] and included age > 6 months; no prior heartworm prophylaxis; absence of bronchopulmonary parasitism; no evidence of cardiovascular or systemic conditions expected to influence respiratory or cardiovascular CT measurements; and no medication administered before blood collection.

Before induction of general anesthesia, all cats underwent thoracic radiography (right lateral and ventrodorsal projections) and routine laboratory testing (complete blood count and renal parameters) to assess clinical stability before anesthesia. Thoracic radiographs were reviewed to exclude overt cardiopulmonary abnormalities.

Written owner consent was obtained for all cats in Group A. For Group B, thoracic CT images were secondarily analyzed with owner consent, as these cats had originally undergone CT for conditions unrelated to *D. immitis* infection. All procedures were performed in accordance with current European legislation on the protection of animals used for scientific purposes.

### 2.2. Serological Test

Blood samples were collected from all cats by jugular, cephalic, or femoral venipuncture. Samples were placed in serum tubes, centrifuged, and the resulting serum was stored at −20 °C until analysis.

All sera were tested for circulating *D. immitis* antigens using a commercial immunochromatographic kit (Uranotest Dirofilaria^®^, UranoVet S.L., Barcelona, Spain), following the manufacturer’s instructions.

In addition, an indirect ELISA (in-house ELISA, UranoVet S.L., Barcelona, Spain) was performed as previously described [14] to detect serum IgG antibodies against *D. immitis*. Briefly, ELISA wells were coated with recombinant *D. immitis* antigen (Di33 protein; 0.5 µg/mL). Serum samples were diluted 1:100 in the sample diluent and incubated in the coated wells. After washing, the conjugate was added, followed by additional washing steps. The reaction was then developed using TMB substrate conjugated with horseradish peroxidase, and stopped with sulfuric acid. Optical density was measured at 450 nm. According to the manufacturer’s guidelines, samples with a cut-off value ≥ 1 were considered seropositive for *D. immitis* antibodies, whereas samples with values < 1 were considered seronegative.

### 2.3. Computed Tomography Analysis

Thoracic CT examinations were performed using a 16-slice helical multidetector CT scanner (Canon Toshiba Astelion, Canon Medical Systems, Tokyo, Japan). Cats were positioned in sternal recumbency with the head extended cranially. Because the respiratory phase can influence bronchial lumen diameter, total bronchial diameter, and pulmonary vascular dimensions, a lack of respiratory phase standardization was considered a potential source of measurement variability. No universally standardized method for controlling the respiratory cycle during thoracic CT acquisition in cats has been established [15]. Therefore, all cats were scanned under the same anesthetic protocol to reduce procedural variability, although no predefined inspiratory breath-hold or standardized respiratory phase was applied.

Premedication consisted of intravenous midazolam (0.2 mg/kg; Midazolam, B. Braun Medical, Barcelona, Spain) and intravenous butorphanol (0.2 mg/kg; Torphadine^®^, Dechra, Northwich, UK). Anesthesia was induced with intravenous propofol (0.6 mg/kg; Propofol Lipuro^®^, B. Braun VetCare, Barcelona, Spain), followed by endotracheal intubation and maintenance with sevoflurane (2.5%; SevoFlo^®^, Zoetis, Louvain-la-Neuve, Belgium). Cats were connected to mechanical ventilation during anesthesia to maintain stable respiration, and physiological parameters were monitored throughout the procedure. However, image acquisition was not synchronized with a predefined respiratory phase, and no standardized inspiratory breath-hold or fixed airway pressure protocol was applied.

A non-ionic iodinated contrast agent (Xenetix^®^, Guerbet, Roissy, France) was administered intravenously at a dose of 600 mgI/kg through a catheter placed in either the left or right cephalic vein. The contrast medium was injected manually at a constant rate before image acquisition, without the use of a power injector, according to the institution’s routine clinical CT protocol. Thoracic CT was first performed without contrast medium, followed by manual intravenous injection of the contrast agent and immediate acquisition of post-contrast images. The exact delay between contrast administration and image acquisition was not standardized. Because this was a clinical study based on routine CT examinations, contrast administration and timing could not be fully standardized. However, all measurements used for the comparative analyses were obtained from post-contrast images and were based on anatomical structural dimensions, including bronchial lumen diameter, total bronchial diameter, and pulmonary artery diameter, rather than on attenuation or perfusion parameters.

Images were acquired with a 1 mm slice thickness (pitch 0.94) and reconstructed using both soft-tissue and bone/lung algorithms. Transverse datasets were used to generate sagittal and dorsal multiplanar reconstructions. Lung window settings (WL −500; WW 1400) were used to assess the pulmonary arteries, bronchial lumen, and total bronchial diameter.

**Figure 1:**
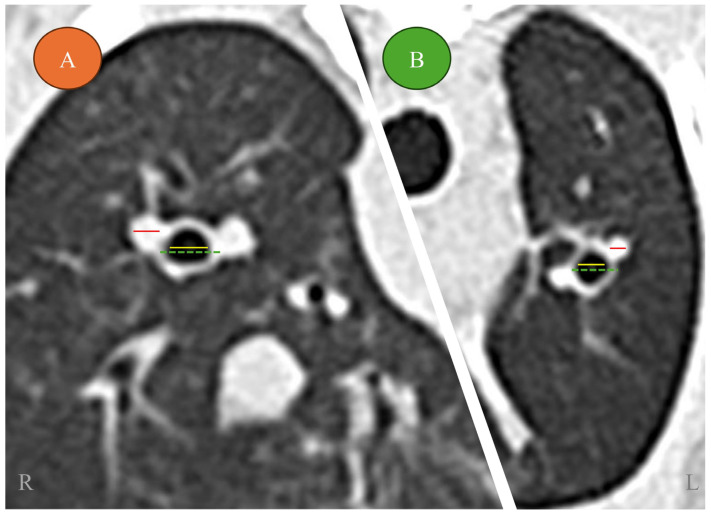
Transverse computed tomography images obtained in two symptomatic cats seropositive for *Dirofilaria immitis* (Group A). From lateral to medial: pulmonary artery (red line), bronchial lumen (yellow line) and total bronchial diameter (green dashed line). (**A**) Right caudal lung lobe at the level of T9-T10. (**B**) Cranial subsegment of the left cranial lung lobe at the level of T4–T5. R: Right, L: Left.

Measurements were performed on the transverse plane according to previously established protocols [11,14] at the following vertebral levels: T4–T5 (cranial subsegment of the left cranial lobe and right cranial lobe), T6–T7 (caudal subsegment of the left cranial lobe), and T9–T10 (left and right caudal lobes). Bronchial lumen diameter, total bronchial diameter, and pulmonary artery diameter were recorded (Figure 1). Thus, a total of five bronchial lumina, five total bronchial diameters, and five pulmonary arteries were evaluated per animal. All measurements were performed on post-contrast images, and only datasets without relevant respiratory motion artifacts and with adequate visualization of the pulmonary arteries and bronchial structures were included in the analysis.

**Figure 2:**
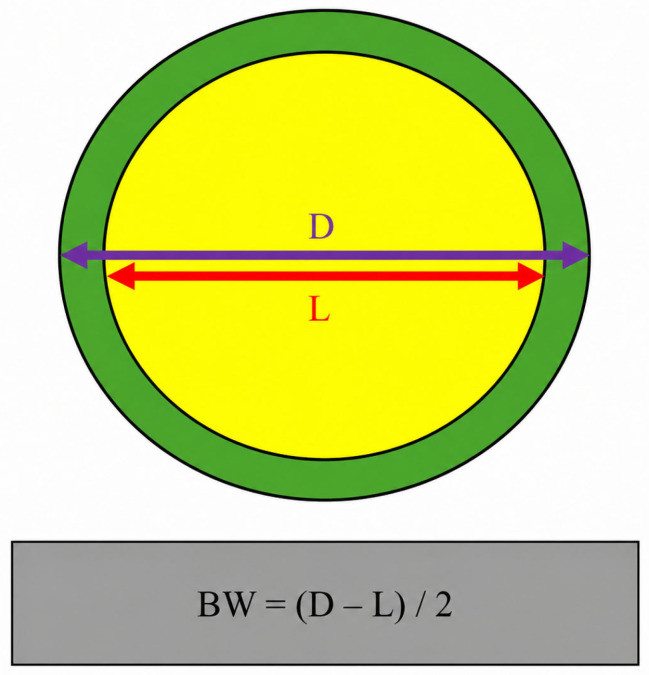
Diagram showing the total bronchial diameter (D; purple arrows) and bronchial lumen diameter (L; red arrow) used to calculate bronchial wall (BW) thickness using the formula shown in the grey box.

Bronchial wall thickness was calculated indirectly from linear measurements using the method described by Won et al. [16], according to the formula BW = (D − L)/2, where D represents total bronchial diameter and L represents bronchial lumen diameter (Figure 2). From these values, BW/B and BW/A ratios were calculated for the cranial and caudal lung lobes, following the previously described protocol [16].

All images were reviewed using Horos software (version 3.0; The Horos Project, Annapolis, MD, USA). All measurements used for the main comparative analyses were obtained by a single primary observer who was blinded to group allocation during image analysis.

### 2.4. Statistical Analysis

Frequencies and percentages were reported for categorical variables. Between-group differences in categorical variables were assessed using Pearson’s chi-square test, and Fisher’s exact test was used when appropriate for 2 × 2 contingency tables. For continuous variables, between-group differences were assessed using the Mann–Whitney U test.

To compare CT-derived measurements and ratios between groups and among lung lobes, repeated-measures mixed linear models (MLMs) adjusted for age and body weight were fitted. A compound symmetry covariance structure was used to account for the within-subject correlation between lung areas, assuming constant variance and equal correlation across areas. The Kenward–Roger method was applied to obtain more reliable inferences given the small sample size.

Multiple comparisons were adjusted using the Bonferroni correction to control type I error. Where applicable, estimated marginal means and between-group differences were reported with 95% confidence intervals (CI) to provide information on the precision of the estimates, particularly given the limited sample size. Effect sizes were reported to aid interpretation. For categorical variables, Cramér’s V was used, with effect sizes classified as negligible (0.00–0.09), low (0.10–0.29), medium (0.30–0.49), and high (>0.50). For continuous variables, the non-parametric effect size was calculated as r = |z|/√(N) with values interpreted as small (0.1–0.3), medium (0.3–0.5), and large (>0.5). Cohen’s d was also reported as a parametric effect size and interpreted as small (0.2–0.4), medium (0.5–0.8), and large (>0.8).

Intra-observer repeatability and inter-observer reproducibility were assessed using the intraclass correlation coefficient (ICC) with an absolute-agreement definition. An absolute agreement ICC was used for both intra-observer and inter-observer analyses. A two-way mixed-effects model was used for intra-observer repeatability, and a two-way random-effects model was used for inter-observer reproducibility. For the reproducibility analysis, a subset of ten CT examinations was selected and independently re-evaluated by a second observer. This reproducibility subset included measurements from the right and left caudal lobes. Both observers were blinded to group allocation during the reproducibility assessment, and the second observer was also blinded to the initial measurements. Each observer repeated the measurements in a separate session to assess intra-observer repeatability, whereas agreement between observers was used to assess inter-observer reproducibility. This assessment was therefore limited to a subset of animals and to two lung regions, rather than encompassing all cats and all evaluated lung regions.

Statistical significance was primarily interpreted at α = 0.05, with *p* < 0.01 considered strong evidence of statistical significance and *p* < 0.10 interpreted as a tendency rather than definitive significance. All analyses were performed using SPSS Statistics for Windows (version 25.0; IBM Corp., Armonk, NY, USA).

## 3. Results

### 3.1. Descriptive Analysis

Of the 27 cats included, 13 (48.1%) were male and 14 (51.9%) were female. Age ranged from 2 to 15 years (mean, 4.17 years), and the mean body weight was 4.0 kg. The most common breed was European Shorthair (23/27; 85.2%). No significant between-group differences were detected for sex (*p* = 0.472), breed (*p* = 0.217), or age (*p* = 0.307). However, body weight differed significantly between groups, with higher values in Group A than in Group B (4.50 kg vs. 3.27 kg, respectively; *p* = 0.034, r = 0.404).

In the descriptive CT measurements, pulmonary artery diameter tended to be larger in Group B. In contrast, bronchial lumen diameter was larger in Group A in all lung lobes except in the caudal subsegment of the left cranial lung lobe, and total bronchial diameter was larger in Group A across all evaluated lobes. These measurements are presented in Table 1 to provide the anatomical context for the derived bronchial wall indices and to allow transparent interpretation of the calculated BW/B and BW/A ratios.

**Figure 3:**
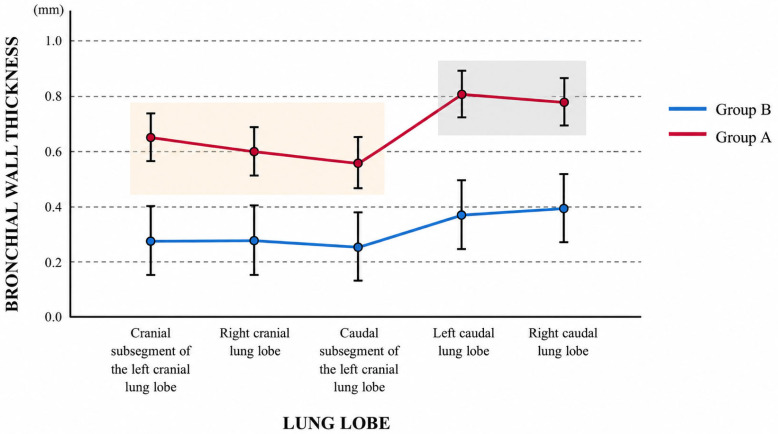
Bronchial wall thickness (mm) across the five evaluated lung regions. Lines represent mean bronchial wall values for Group A (*Dirofilaria immitis* antibody-seropositive cats with lower-airway clinical signs) and Group B (asymptomatic seronegative comparison cats), and vertical bars represent the 25th and 75th percentiles. Cranial regions are highlighted by the orange box, and caudal lobes by the grey box. Group A showed higher bronchial wall values than Group B across all evaluated lung regions. Effect sizes for between-group differences ranged from d = 0.5 to d = 1.0.

Bronchial wall thickness, calculated from bronchial lumen and total bronchial diameter measurements, was significantly higher in Group A in every lobe (*p* < 0.05; d = 1.2–1.7). The estimated between-group differences ranged from 0.275 (95% CI: 0.152–0.397) to 0.653 (95% CI: 0.575–0.731) mm for cranial subsegment of the left cranial lobe, from 0.279 (95% CI: 0.156–0.401) to 0.601 (95% CI: 0.523–0.679) mm for right cranial lobe, from 0.253 (95% CI: 0.131–0.375) to 0.558 (95% CI: 0.48–0.636) mm for caudal subsegment of the left cranial lobe, from 0.368 (95% CI: 0.246–0.491) to 0.81 (95% CI: 0.732–0.888) mm for left caudal lobe, and from 0.396 (95% CI: 0.273–0.518) to 0.78 (95% CI: 0.703–0.858) mm for right caudal lobe. Within Group A, bronchial wall values were relatively homogeneous across cranial and across caudal lobes, with significantly greater bronchial wall values in the caudal lobes than in the cranial lobes (Figure 3). The left and right caudal lobes showed similar bronchial wall values, which were consistently higher in seropositive cats (mean difference [95% CI] between seropositive and seronegative cats: 0.442 (95% CI: 0.293–0.591) for the left caudal lobe and 0.385 (95% CI: 0.236–0.534) for the right caudal lobe; *p* < 0.001 for both). No significant inter-lobar differences were observed in Group B. However, the overall pattern of differences between lung regions did not differ significantly between groups (Area × Group interaction: F(4100) = 0.9445; *p* = 0.441), indicating a similar pattern of variation (parallel lines and same structure). Given the limited sample size, particularly in Group B, this finding should be interpreted as an absence of statistically detected interaction rather than definitive evidence of identical regional patterns between groups.

### 3.2. Comparison of BW/B and BW/A Ratios Based on the ELISA Results

**Figure 4:**
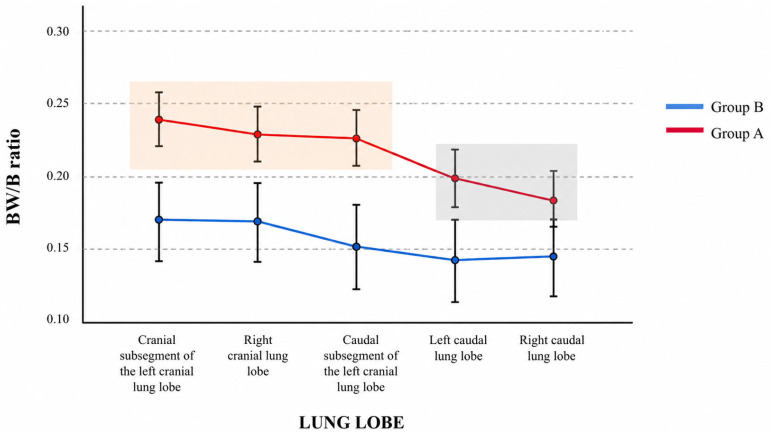
Bronchial wall-to-bronchus ratio (BW/B; unitless) across the five evaluated lung regions in Group A (*Dirofilaria immitis* antibody-seropositive cats with lower-airway clinical signs) and Group B (asymptomatic seronegative comparison cats). Lines represent mean BW/B values for each group, and vertical bars represent the 25th and 75th percentiles. Cranial regions are highlighted by the orange box, and caudal lobes by the grey box. Group A showed higher BW/B values than Group B across all evaluated lung regions. Effect sizes for between-group differences ranged from d = 0.6 to d = 1.1.

A total of 135 BW/B and BW/A ratio sets were calculated across all lung lobes in Group A and Group B. Mean BW/B ratio values were significantly higher in Group A than in Group B in all lung lobes (*p* < 0.05; 0.7 < d < 1.5), as illustrated in Figure 4 by the consistently higher curve for Group A. The estimated between-group differences ranged from 0.171 (95% CI: 0.144–0.198) to 0.239 (95% CI: 0.222–0.256) mm for cranial subsegment of the left cranial lobe, from 0.17 (95% CI: 0.143–0.197) to 0.229 (95% CI: 0.211–0.246) mm for right cranial lobe, from 0.153 (95% CI: 0.125–0.18) to 0.227 (95% CI: 0.209–0.244) mm for caudal subsegment of the left cranial lobe, from 0.143 (95% CI: 0.116–0.17) to 0.201 (95% CI: 0.183–0.218) mm for left caudal lobe, and from 0.147 (95% CI: 0.119–0.174) to 0.184 (95% CI: 0.167–0.201) mm for right caudal lobe.

Within Group A, mean BW/B values did not differ significantly among the right cranial and left cranial regions (cranial and caudal subsegments), nor between the left and right caudal lobes. Nevertheless, BW/B values in the caudal lobes (grey box) were lower than those in the cranial lobes (orange box), resulting in two clearly distinct subgroups (cranial vs. caudal). These differences reached the predefined tendency threshold of *p* < 0.10 for comparisons between the right and left caudal lobes and between the left caudal lobe and the caudal subsegment of the left cranial lobe. In contrast, no statistically significant differences among lung areas were detected in Group B.

Although cranial–caudal differences were visually more evident in Group A than in Group B, the Area × Group interaction was not significant (F(4100) = 1.091; *p* = 0.365), indicating that the pattern of BW/B variation across lung areas was broadly similar between groups. Given the small number of seronegative comparison cats, this result should be interpreted cautiously and does not exclude the possibility of subtle regional differences that the study was unable to detect.

**Figure 5:**
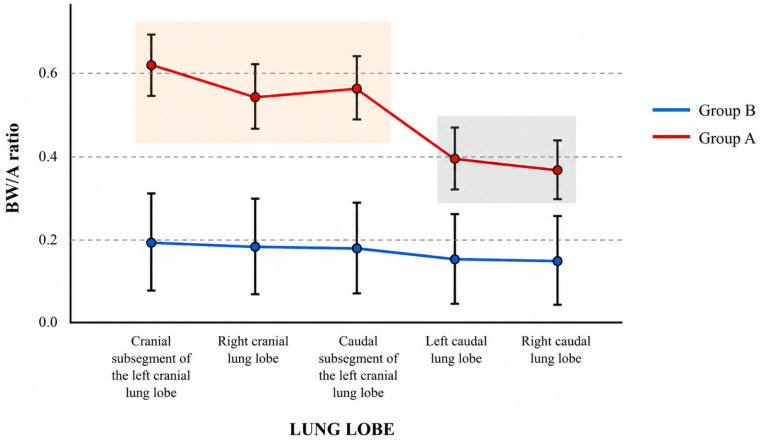
Bronchial wall–to–pulmonary artery ratio (BW/A; unitless) across the five evaluated lung regions in Group A (*Dirofilaria immitis* antibody-seropositive cats with lower-airway clinical signs) and Group B (asymptomatic seronegative comparison cats). Lines represent mean BW/A values for each group, and vertical bars represent the 25th and 75th percentiles. Cranial regions are highlighted by the orange box, and caudal lobes by the grey box. Group A showed higher BW/A values than Group B across all evaluated lung regions. Effect sizes for between-group differences ranged from d = 0.6 to d = 1.0.

For the BW/A ratio, mean values were also significantly higher in Group A across all lobes (*p* < 0.05; 1.0 < d < 1.9) (Figure 5). The estimated between-group differences ranged from 0.192 (95% CI: 0.083–0.301) to 0.626 (95% CI: 0.557–0.696) mm for cranial subsegment of the left cranial lobe, from 0.18 (95% CI: 0.071–0.289) to 0.552 (95% CI: 0.482–0.621) mm for right cranial lobe, from 0.177 (95% CI: 0.068–0.286) to 0.572 (95% CI: 0.503–0.642) mm for caudal subsegment of the left cranial lobe, from 0.154 (95% CI: 0.045–0.263) to 0.414 (95% CI: 0.344–0.483) mm for left caudal lobe, and from 0.148 (95% CI: 0.039–0.257) to 0.382 (95% CI: 0.313–0.452) mm for right caudal lobe. In this case, the Area × Group interaction was significant (F(4100) = 3.229; *p* = 0.015), indicating that the pattern of differences between cranial (orange box) and caudal (grey box) areas differed between Group A and Group B (i.e., non-parallel profiles with different structures). Therefore, the distribution of BW/A values across lung areas in Group A was distinct from that observed in Group B in this cohort. However, given the limited sample size, this interaction should be interpreted as an exploratory finding requiring confirmation in larger datasets.

### 3.3. Intra- and Inter-Observer Reproducibility

For intra-observer comparisons (both observers), ICC values for the six evaluated parameters were excellent for both observers, exceeding 0.85 for the left caudal lobe and 0.90 for the right caudal lobe (Table 2).

Inter-observer agreement was very high for all parameters, except for the pulmonary artery measurement in the right caudal lobe, where the ICC was below 0.80 (*p* < 0.05) (Table 3).

## 4. Discussion

HARD is associated with exposure to immature stages of *D. immitis* that reach the pulmonary circulation and trigger inflammatory lesions involving the pulmonary vasculature, parenchyma, and lower airways [1]. Histopathological studies have consistently described inflammatory infiltrates, irregular interstitial myofibrosis, and peribronchial wall thickening, providing a mechanistic basis for the predominance of cough, tachypnea, and respiratory distress observed in affected cats [1,4,6,10]. Radiographically, these lesions often appear as diffuse or regional bronchointerstitial patterns, underscoring bronchial involvement as a key feature of HARD [17]. In the present study, cats with lower-airway clinical signs and *D. immitis* antibody seropositivity showed increased CT-derived bronchial wall thickness, BW/B, and BW/A ratios compared with seronegative comparison cats. Importantly, antibody seropositivity indicates exposure and immune response but does not confirm that immature *D. immitis* stages were present in the pulmonary vasculature at the time of CT examination. Therefore, the cats in Group A should be interpreted as having a clinical and serological profile compatible with HARD, rather than confirmed active HARD, and the CT abnormalities described here should be interpreted as quantitative indicators of bronchial remodeling in cats with a clinical and serological profile compatible with HARD, rather than imaging features proven to be specific for this syndrome.

CT offers relevant advantages over conventional radiography, as it eliminates superimposition and allows detailed evaluation of bronchial and vascular structures. Previous CT studies in feline heartworm disease, particularly in cats infected with adult *D. immitis*, have mainly focused on vascular and luminal bronchial changes, including pulmonary arterial enlargement, tortuosity, thromboembolic lesions, and bronchial–vascular relationships [1,6,11,12,13]. In contrast, only a small number of studies have explored quantitative CT indices in cats compatible with HARD, in which prior quantitative CT work suggested that airway remodeling may predominate over vascular luminal changes during early or immature stages of feline heartworm infection [14]. The present study extends this approach by specifically evaluating bronchial wall-focused indices, which may better capture structural airway remodeling than lumen-based measurements alone.

To the authors’ knowledge, this study is the first to specifically evaluate CT-derived bronchial wall indices in cats with clinical and serological profiles compatible with HARD. Both absolute bronchial wall thickness and the normalized ratios BW/B and BW/A were significantly increased in seropositive cats with lower-airway clinical signs compared with asymptomatic seronegative cats across all evaluated lung regions, supporting the presence of diffuse bronchial wall involvement in this cohort. These findings are consistent with histopathological descriptions of peribronchial thickening and fibrosis reported in naturally and experimentally infected cats [2], extending prior CT-based work by incorporating bronchial wall-focused measurements rather than relying exclusively on luminal relationships. However, because no respiratory disease comparator group was included, these results should not be interpreted as demonstrating that such CT abnormalities are specific to HARD. Bronchial wall thickening is not expected to be specific to this syndrome, and similar CT-derived changes may occur in other feline inflammatory airway diseases, including asthma and chronic bronchitis. Therefore, the increased BW/B and BW/A ratios observed in this study should be interpreted as quantitative descriptors of bronchial wall remodeling within a HARD-compatible cohort, not as criteria capable of distinguishing HARD from other respiratory disorders.

Among the evaluated indices, the BW/A ratio showed marked differences between cats with a HARD-compatible profile and seronegative comparison cats, especially in the cranial lung lobes. One possible explanation is that BW/A may be less affected by concomitant bronchial lumen dilation than BW/B because BW/B uses the bronchial diameter itself as the denominator, whereas an increase in bronchial wall thickness combined with a comparatively stable pulmonary arterial diameter, expected in HARD-compatible cats and as observed in this cohort, results in a marked elevation of the BW/A ratio, supporting its potential value as an exploratory quantitative descriptor of bronchial involvement. In the present cohort, bronchial lumen dilation was more evident in the caudal lung lobes, consistent with prior CT observations in cats compatible with HARD [14]. When bronchial wall thickness and bronchial luminal diameter increase in parallel, BW/B may partially normalize wall thickening, thereby reducing its sensitivity to detect regional differences. This mechanism may explain why BW/B showed a lower discriminatory capacity than BW/A in certain lobular regions. In contrast, BW/A may provide a complementary description of bronchial wall remodeling because it relates wall thickness to the adjacent pulmonary artery rather than to the bronchial lumen. Nevertheless, this interpretation remains hypothesis-generating and requires validation in larger cohorts.

The distribution of the ratios also differed according to lung area. BW/B showed higher mean values in the cranial lung lobes; however, the absence of a significant area-by-group interaction indicates that this pattern cannot be considered specific to affected cats. In contrast, BW/A was the only index that showed a significant area-by-group interaction, suggesting that regional variation in this ratio may differ between cats with a HARD-compatible profile and seronegative comparison cats. This suggests that BW/A may be particularly sensitive to subtle regional differences in bronchial remodeling that are less evident when ratios based solely on bronchial dimensions are used. Comparable CT-based studies in feline asthma have reported diffuse bronchial wall involvement without clear cranial or caudal predominance, whereas studies in dogs with chronic bronchitis have described higher bronchial wall-related indices in the cranial lung lobes. Therefore, the higher BW/A values observed in cranial lobes may represent a cohort-specific or disease-related pattern of airway remodeling rather than generalized lobar susceptibility; however, this interpretation should be considered preliminary.

Immature *D. immitis* stages have been reported to lodge preferentially in smaller, more distal pulmonary arteries, particularly in caudal lung regions, and a substantial proportion appear to die shortly after arrival, likely facilitated by a strong local immune response [18,19]. This caudal vascular predilection provides a useful framework for interpreting regional findings. If immature parasites or their inflammatory and embolic sequelae initially affect the caudal pulmonary vasculature to a greater extent, caudal lung regions may experience a stronger or earlier inflammatory milieu and ventilation–perfusion disturbance, which could contribute to changes in bronchial caliber in those lobes. In turn, such caudal bronchial lumen dilation would be expected to disproportionately influence bronchus-based ratios (BW/B), potentially attenuating their ability to reflect wall thickening in caudal regions, precisely the effect observed here. Conversely, the higher BW/A values observed in cranial lobes may reflect the combination of bronchial wall remodeling and relatively less pronounced cranial vascular luminal change, yielding higher wall-to-artery ratios in those regions. Overall, these preliminary patterns raise the hypothesis that airway remodeling in cats compatible with HARD may not simply mirror the classic caudal predominance described for pulmonary vascular involvement in heartworm disease but may instead reflect a more complex, regionally heterogeneous interaction between airway inflammation/remodeling and vascular involvement. Further studies incorporating standardized respiratory phase acquisition and, when possible, tissue correlation will be needed to determine whether this cranial predominance of BW/A represents a reproducible feature of HARD or is influenced by cohort- and protocol-specific factors.

CT-derived bronchial wall indices are well established in human medicine as markers of airway remodeling in chronic inflammatory diseases such as asthma and chronic obstructive pulmonary disease, where wall thickening reflects subepithelial fibrosis, smooth muscle hypertrophy, increased vascularity, and mucus-related changes [20,21]. Similar methodologies have also been validated in veterinary medicine, including dogs with chronic bronchitis and related airway disorders, and in clinically asthmatic cats, where BW/A and BW/B ratios have shown good diagnostic performance and agreement [16,22,23,24]. The fact that similar indices are altered in non-parasitic inflammatory airway diseases reinforces that the CT-derived changes observed in the present study should not be interpreted as disease-specific. Rather, they represent objective indicators of bronchial remodeling that may occur in different inflammatory lung diseases. In cats with a HARD-compatible profile, these alterations may reflect the inflammatory response associated with exposure to immature stages of *D. immitis*.

From a clinical perspective, and pending validation in larger comparative cohorts, quantitative assessment of bronchial wall remodeling may add complementary information to traditional thoracic imaging and to previously proposed luminal and bronchial–vascular relationship indices. While radiography can suggest bronchointerstitial disease, CT-based wall indices provide objective, standardized measurements of airway wall involvement that may be useful for describing the extent and distribution of bronchial remodeling when conventional findings are subtle or nonspecific. In endemic areas—where exposure to *D. immitis* is common and HARD may remain underrecognized—these indices may contribute to the overall imaging characterization of cats with compatible clinical and serological findings, but they should not be interpreted in isolation or as diagnostically specific for HARD [9]. They may also serve as exploratory quantitative endpoints in future longitudinal studies evaluating disease evolution or response to interventions, although prospective comparative validation is required before any clinical thresholds, diagnostic applications, or prognostic implications can be established.

The present study should be regarded as a preliminary exploratory investigation, and several limitations should be acknowledged. First, the sample size was modest, particularly in the seronegative comparison group, which limits statistical power and reduces the ability to detect subtle lobar differences or area-by-group interaction effects. Therefore, non-significant interaction terms should not be interpreted as definitive evidence of absence of regional differences between groups. In addition, the small sample size may have widened the uncertainty around effect estimates, as reflected by the 95% confidence intervals. Accordingly, the observed differences and regional patterns should be considered preliminary and should be confirmed in larger cohorts.

Second, the study population limits disease-specific interpretation. The control cats were clinically asymptomatic for cardiorespiratory disease and seronegative for *D. immitis*, but they were not healthy volunteers, as shown by the CT for unrelated clinical indications, where the images were secondarily analyzed. Although strict inclusion criteria were applied, thoracic radiographs were reviewed, and overt cardiopulmonary disease was excluded, this clinically selected control population may not fully represent a healthy baseline population. Therefore, residual confounding or subclinical airway abnormalities cannot be completely ruled out. In addition, no comparator group of cats with respiratory disease but negative *D. immitis* serology was included. Consequently, the increased BW/B and BW/A ratios cannot be considered specific to HARD and may instead reflect bronchial remodeling shared with other feline inflammatory airway diseases, such as asthma or chronic bronchitis.

Third, classification as HARD-compatible relied on respiratory signs and antibody seropositivity. In clinical practice, HARD is usually suspected based on a combination of clinical, serological, and imaging findings, rather than a single definitive ante-mortem test. Because antibodies reflect exposure rather than definitive active infection, antibody status does not allow for the determination of the timing of exposure or the current stage of disease activity. Consequently, cats in Group A may have represented different biological phases, ranging from more acute inflammatory responses to more chronic airway remodeling. This heterogeneity may have influenced the magnitude and distribution of the CT-derived bronchial wall indices.

Fourth, histopathological confirmation was not available, preventing direct correlation between CT-derived bronchial wall indices and tissue-level lesions such as peribronchial inflammation, fibrosis, smooth muscle changes, or other remodeling processes. Therefore, although the CT findings are consistent with previously described pathological changes in feline Heartworm-Associated Respiratory Disease, the specific histological substrate of the increased BW/B and BW/A ratios could not be confirmed in this cohort. Such confirmation is generally limited to post-mortem studies or experimental infection models.

Fifth, CT acquisition was performed without standardized inspiratory pressure or predefined respiratory phase control, which represents an important methodological limitation. The respiratory phase can influence bronchial lumen diameter, total bronchial diameter, apparent bronchial wall thickness, and pulmonary artery diameter; consequently, it may also affect the derived BW/B and BW/A ratios. Although all cats were examined under the same anesthetic protocol, were mechanically ventilated, and only datasets without relevant respiratory motion artifacts were included, interindividual differences in lung inflation or respiratory phase at the time of acquisition cannot be excluded. These differences may have contributed to measurement variability and may have influenced the magnitude of between-group or regional differences. Future studies should incorporate standardized respiratory control whenever feasible, such as acquisition at a predefined respiratory phase, inspiratory breath-hold protocols, controlled ventilation with fixed airway pressure, or other standardized lung inflation techniques, in order to improve measurement reproducibility and reduce respiratory-phase-related variability.

Sixth, contrast administration and acquisition timing were not standardized, as CT examinations followed the institution’s routine clinical protocol and contrast medium was administered manually without a power injector or fixed delay. Because the evaluated variables were anatomical structural dimensions rather than attenuation- or perfusion-based parameters, they are expected to be relatively less dependent on contrast timing. Nevertheless, differences in vascular enhancement may have influenced pulmonary artery boundary definition, potentially contributing to variability in pulmonary artery measurements and, consequently, in the BW/A ratio. By contrast, bronchial wall thickness and BW/B are less directly dependent on vascular enhancement, although image quality and structure delineation remain relevant for all measurements. Future prospective studies evaluating vascular-based CT indices should incorporate standardized contrast injection and acquisition timing whenever feasible.

Finally, all measurements used for the main comparative analyses were obtained by a single primary observer, although this observer was blinded to group allocation during image analysis. While this approach reduced inter-observer variability in the main dataset, it does not account for the full range of variability that may occur across observers in routine or multicenter settings. Intra-observer repeatability and inter-observer reproducibility were high overall; however, these analyses were performed in a limited subset of ten CT examinations and only included selected lung regions. Therefore, although the reliability results support the technical feasibility of the measurement approach, broader validation involving multiple observers, all evaluated lung regions, larger datasets, and ideally multiple centers would further strengthen generalizability.

## 5. Conclusions

In conclusion, in this preliminary cohort, cats with lower-airway clinical signs and *D. immitis* antibody seropositivity compatible with HARD showed significant and diffuse bronchial wall remodeling detectable by CT. Both BW/B and BW/A were increased across lung regions, and BW/A was the only index showing a significant area-by-group interaction, suggesting that it may be a promising exploratory descriptor of bronchial wall remodeling in this clinical setting. However, these findings should be interpreted cautiously, as similar bronchial changes may also occur in other feline airway diseases, and the diagnostic specificity of these CT-derived indices remains uncertain. Therefore, quantitative CT assessment of the bronchial wall may represent a useful complementary approach for characterizing airway involvement in cats with clinical and serological profiles compatible with HARD, but it does not represent a disease-specific diagnostic tool nor a basis for differential diagnosis in the absence of appropriate comparator groups. Future studies should aim to validate these preliminary findings in larger comparative prospective cohorts, including healthy controls and cats with non-heartworm-related respiratory disease, incorporate standardized respiratory phase control where feasible, and explore correlations with clinical severity, longitudinal outcomes, and, when available, histopathological changes.

## Figures and Tables

**Table 1:** Descriptive computed tomography (CT) measurements of pulmonary artery diameter, bronchial lumen diameter, and total bronchial diameter by lung region and study group. Values are reported as mean, standard deviation (SD), and median [25th–75th percentiles], expressed in millimeters. Group A included cats with lower-airway clinical signs and *Dirofilaria immitis* antibody seropositivity compatible with HARD; Group B included asymptomatic seronegative comparison cats. Statistical comparisons refer to between-group differences within each lung region. Legend: PA, pulmonary artery. Significance levels: + *p* < 0.10; * *p* < 0.05; ** *p* < 0.01; *** *p* < 0.001.

Lung Lobe	Variable	Groups	Results of the CT Scan According to Results of the ELISA Technique				*p*-Value Mann–Whitney	Effect Size r
			Valid N	Mean (mm)	SD	Median [25th and 75th Percentiles] (mm)		
Left cranial (cranial subsegment)	PA	Group A	19	1.11	0.27	1.12 [0.89–1.27]	0.029 *	0.482
		Group B	8	1.43	0.35	1.32 [1.17–1.69]		
	Bronchial lumen	Group A	19	1.47	0.46	1.28 [1.16–1.80]	0.019 *	0.449
		Group B	8	1.05	0.31	0.91 [0.83–1.26]		
	Bronchial diameter	Group A	19	2.78	0.54	2.87 [2.32–3.25]	<0.001 ***	0.716
		Group B	8	1.60	0.42	1.40 [1.30–1.90]		
Left cranial (caudal subsegment)	PA	Group A	19	1.00	0.29	1.01 [0.79–1.17]	0.066 ^+^	
		Group B	8	1.67	0.97	1.46 [0.94–2.19]		
	Bronchial lumen	Group A	19	1.36	0.47	1.21 [1.02–1.68]	0.360	
		Group B	8	1.27	0.75	1.02 [0.71–1.62]		
	Bronchial diameter	Group A	19	2.48	0.82	2.23 [1.80–3.31]	0.066 ^+^	
		Group B	8	1.77	0.88	1.47 [1.10–2.20]		
Left caudal	PA	Group A	19	2.08	0.53	2.04 [1.60–2.34]	0.066 ^+^	
		Group B	8	2.36	0.15	2.33 [2.26–2.39]		
	Bronchial lumen	Group A	19	2.47	0.55	2.42 [2.08–2.75]	<0.001 ***	0.613
		Group B	8	1.76	0.17	1.81 [1.63–1.91]		
	Bronchial diameter	Group A	19	4.09	0.65	4.06 [3.74–4.43]	<0.001 ***	0.776
		Group B	8	2.50	0.33	2.45 [2.35–2.79]		
Right cranial	PA	Group A	19	1.16	0.31	1.20 [0.86–1.39]	0.058 ^+^	
		Group B	8	1.57	0.49	1.43 [1.18–2.09]		
	Bronchial lumen	Group A	19	1.47	0.40	1.59 [1.11–1.75]	0.022 *	0.439
		Group B	8	1.08	0.36	0.96 [0.80–1.32]		
	Bronchial diameter	Group A	19	2.67	0.54	2.77 [2.41–3.14]	<0.001 ***	0.654
		Group B	8	1.63	0.49	1.43 [1.30–2.00]		
Right caudal	PA	Group A	19	2.16	0.64	2.19 [1.81–2.48]	0.075 ^+^	
		Group B	8	2.65	0.71	2.70 [2.18–3.00]		
	Bronchial lumen	Group A	19	2.73	0.73	2.86 [2.37–3.15]	0.009 **	0.490
		Group B	8	1.92	0.63	1.92 [1.46–2.42]		
	Bronchial diameter	Group A	19	4.29	0.98	4.25 [3.76–4.60]	<0.001 ***	0.643
		Group B	8	2.71	0.82	2.69 [2.18–3.27]		

**Table 2:** Intra-observer repeatability analysis for CT-derived measurements (both observers). Legend: ICC, intraclass correlation coefficient; PA, pulmonary artery; (***) *p* < 0.001.

Reliability Analysis	Lung Lobe	Variable	ICC
Intra-observer 1	Left caudal	PA	0.874 ***
		Bronchial lumen	0.877 ***
		Bronchial diameter	0.960 ***
	Right caudal	PA	0.920 ***
		Bronchial lumen	0.968 ***
		Bronchial diameter	0.976 ***
IntrA-observer 2	Left caudal	PA	0.873 ***
		Bronchial lumen	0.888 ***
		Bronchial diameter	0.945 ***
	Right caudal	PA	0.963 ***
		Bronchial lumen	0.959 ***
		Bronchial diameter	0.978 ***

**Table 3:** Inter-observer reproducibility analysis for CT-derived measurements. Legend: ICC, intraclass correlation coefficient; PA, pulmonary artery; (*) *p* < 0.05, (**) *p* < 0.01, (***) *p* < 0.001.

Reliability Analysis	Lung Lobe	Variable	ICC
IntER-observeR	Left caudal	PA	0.998 ***
		Bronchial lumen	0.999 ***
		Bronchial diameter	1.000 ***
	Right caudal	PA	0.737 *
		Bronchial lumen	0.939 **
		Bronchial diameter	1.000 ***

## Data Availability

The raw data supporting the conclusions of this article will be made available by the authors, without undue reservation.

## Abbreviations

The following abbreviations are used in this manuscript:

BW	Bronchial wall
BW/A	Bronchial wall thickness/Pulmonary artery
BW/B	Bronchial wall thickness/Bronchial diameter
CI	Confidence intervals
CT	Computed Tomography
HARD	Heartworm-Associated Respiratory Disease
ICC	Intraclass correlation coefficient
PA	Pulmonary artery
